# Comparing the Immunogenicity of AS03-Adjuvanted 2009 Pandemic H1N1 Vaccine with Clinical Protection in Priority Risk Groups in England

**DOI:** 10.1371/journal.pone.0056844

**Published:** 2013-02-22

**Authors:** Chee-Fu Yung, Nick Andrews, Katja Hoschler, Elizabeth Miller

**Affiliations:** 1 Department of Clinical Epidemiology, Communicable Disease Centre, Tan Tock Seng Hospital, Singapore, Singapore; 2 Immunisation, Hepatitis and Blood Safety Department, Health Protection Services, Colindale, Health Protection Agency, London, United Kingdom; 3 Statistics Unit, Health Protection Services, Colindale, Health Protection Agency, London, United Kingdom; 4 Respiratory Virus Unit, Virus Reference Department, Microbiology Services, Colindale, Health Protection Agency, London, United Kingdom; Centers for Disease Control and Prevention, United States of America

## Abstract

In England, during pandemic 2009 H1N1, vaccine efficacy and immunogenicity population studies in priority groups were rolled out in parallel to evaluate the pandemic vaccination programme. This provided a unique opportunity to compare immunogenicity and clinical protection in the same population and thus provide insights into the correlates of protection for the pandemic H1N1 2009 vaccine in risk groups. While clinical protection from AS03-adjuvanted pandemic 2009 H1N1 vaccine was high in those aged <25 years and pregnant women, effectiveness in older adults with chronic conditions has been found to be surprisingly poor. Here we present results from the immunogenicity study derived from the same population. Individuals from priority groups eligible for pandemic vaccination attending participating general practices were recruited. Pre and post-vaccination blood samples were collected and HI antibody testing to assess immune response to vaccination performed. The final cohort consisted of 610 individuals: 60 healthy children aged <5 years; 32 healthy pregnant women; 518 individuals from risk groups. Seroconversion rate in healthy children aged <5 years (87%, 95% CI: 75% to 94%) was higher than that of risk groups combined (65%, 95% CI: 61% to 69%) (p<0.001). Multivariable analysis of risk groups showed that the size of response in those who did seroconvert was lower in those who received the 2009/10 seasonal TIV (Fold effect: 0.52, 0.35 to 0.78). Predicted immunological boosting from higher pre-vaccine titres after 2009 pandemic H1N1 vaccination only occurred in children (seroconversion rate = 92%) and not in individuals aged 10 to 39 from risk groups (seroconversion rate = 74%). The lack of clinical protection identified in the same population in older adults from risk groups could be attributed to these lower seroresponses. Current immunogenicity licensing criteria for pandemic influenza vaccine may not correlate with clinical protection in individuals with chronic disease or immunocompromised.

## Introduction

The clinical impact of the H1N1 (2009) pandemic influenza virus was generally mild except for those with underlying conditions such as pregnancy or co-morbidities already recognised as risk factors for severe seasonal influenza. [1] Targeting these high risk groups with pandemic influenza vaccination was shown to be the most cost effective strategy for mitigating the clinical consequences of the pandemic and informed the vaccination policy in the United Kingdom. [2] However, at the time decisions about vaccine prioritisation were made immunogenicity data for the monovalent 2009 pandemic H1N1 strain vaccine in high risk groups was unavailable as pre-licensure clinical trials had focussed on healthy individuals. Compared to the general population, high risk individuals, such as those with chronic disease or immunosuppression, may have different antibody responses due to their clinical condition. They are also likely to have received seasonal trivalent influenza vaccine (TIV) in previous years which has been known to affect immune responses in previous seasonal TIV studies although the impact on clinical protection is unclear. [3] Recent work has demonstrated that seasonal TIV can reduce the immunogenicity of the H1N1 (2009) pandemic strain vaccine in children and healthy adults. [4], [5] In these cohorts, clinical protection studies have not shown any significant loss of efficacy. [6], [7].

In the United Kingdom, vaccination with an oil in water adjuvanted vaccine (Pandemrix™) began in late October 2009 and pregnant women as well as individuals with chronic conditions identified as risk factors for severe seasonal influenza were prioritized [8]. As from January 2010, vaccination was recommended for all children aged under 5 years of age. [9] Andrews at al have reported the effectiveness of the vaccine against laboratory confirmed influenza in these high risk groups and children under 5 years of age in England. [6] While protection from a single dose was high in those aged under 25 years and pregnant women, effectiveness in older adults with chronic conditions was surprisingly poor. Similar findings were recently reported from Denmark for the same adjuvanted vaccine. [10].

In view of lack of immunogenicity data for the pandemic vaccine in high risk groups such as those with chronic disease prioritized for vaccination, we conducted an immunogenicity study in parallel with the roll out of the vaccine in England. This provided a unique opportunity to compare immunogenicity and clinical protection in the same population and thus provide insights into the correlates of protection for the monovalent 2009 pandemic H1N1 strain vaccine in high risk groups and young children.

## Materials and Methods

### Ethics Statement

The National Research Ethics Service classified the study as service evaluation and therefore did not require formal ethical approval. Written informed consent was obtained from individuals or parents/guardian.

### Participants

The cohort comprised patients registered with general practices in Hertfordshire and Gloucestershire, England, who were eligible to receive pandemic influenza vaccination as per Department of Health (DH) policy. [8], [9] Individuals attending their practices to receive pandemic influenza vaccination were offered the clinical service option to have two blood tests to check their immune response to the vaccine. The antibody response to vaccination was reported back to the participants as well as their GPs.

### Covariates Collected

Information on the following was collected for inclusion in the analysis: age, sex, clinical condition, previous seasonal vaccination from GP medical records (2007/8, 2008/9, and 2009/10), ethnicity, month of sample collection, date(s) of pandemic vaccine administration and the interval between vaccination and blood collection. Individuals were classified as unvaccinated if there was no documented evidence of previous seasonal vaccination in their GP medical records. At the time the post-vaccination blood was taken, individuals were also asked whether they had experienced an influenza like illness (ILI) since vaccination and if so, the date of onset and results of laboratory confirmation if a swab had been taken for detection of H1N1 (2009) infection.

### Vaccine and Vaccination Schedules

The oil-in water ASO3_A_ adjuvanted pandemic vaccine (Pandemrix™) used in this study was supplied by DH as part of the national pandemic vaccination programme. Doses and schedules followed national recommendations provided to GPs. [8], [9] The dosage of Pandemrix™ recommended for all children aged from six months of age to less than 10 years of age was one half dose (0.25 ml) and for individuals aged 10 years and over, was one dose (0.5 ml). Immunocompromised individuals were recommended to receive two doses.

### Blood Samples and Laboratory Testing

Clotted blood samples were taken mostly at the time of vaccination (latest not more than 48 hours post vaccination) and at least 14 days after the last dose. Haemagglutination Inhibition (HI) antibody testing was carried out using A/California/7/2009-like H1N1 egg-grown reverse-genetics NIBRG-121 virus strain (supplied by the National Institute for Biological Standards and Controls, UK) to measure responses to pandemic H1N1 (2009) viruses. Assays from anonymised individuals were performed at the Respiratory Virus Unit at Health Protection Agency – Colindale according to standard protocols. Briefly, 0.5% turkey red blood cells in PBS and four Haemagglutinating Units (HAU) were used and sera treated with Receptor Destroying Enzyme (RDE manufactured by Denka Seiken Co., Ltd, Japan used according to manufacturer’s recommendation) were tested at an initial dilution of 1∶8 and given serial two-fold dilutions to determine end-point titres; those that were negative were assigned a titre of 1∶4. Specimens were tested in duplicate, and the geometric mean values were used in analyses. Suitable positive and negative laboratory control sera were included in each assay.

### Statistical Methods

To estimate the overall proportion seroconverting with good precision (95% confidence interval width within +/−7% a minimum of 200 vaccinated individuals are required. Only individuals with HI results for paired pre and post vaccination samples were included in the analysis. Age groups chosen to give reasonable numbers in each group were <5, 10–39, 40–54, 55–64, 65+ years of age. Outcome variables were the proportion achieving a ≥4 fold rise in titre to at least 1∶32 (seroconversion rate), the proportion with a post-vaccination HI titre of ≥1∶32 (seroprotection rate), the post vaccination titre and the fold change (ratio post:pre) in titre (seroresponse). Proportions of seroprotected and seroconverting individuals are given along with post vaccination geometric mean titres (GMT) and geometric fold ratios (GMFR) for which 95% confidence intervals are also given. GMFR are also calculated within those who seroconvert. Multivariable logistic regression and normal errors regression was used to assess the independent effects of different factors on seroconversion rate and logged- fold ratios. Model selection was based on retaining significant as well as known important variables.

## Results

In total, 618 individuals provided pre and post vaccination blood samples in the period from November 2009 to March 2010. Of these, only four subjects aged 5–9 years provided paired samples for the analysis. As this age group received the lower vaccine dose they were dropped from further analysis; all four responded to vaccine. In the age group<5 years, only four children had chronic conditions - three of them responded. These four were also dropped from the analyses.

The final sample for analysis consisted of 610 individuals stratified into 4 priority groups (healthy children aged <5 years; healthy pregnant women; those in a risk group with one dose and those with two doses) - their variables are summarised in Table 1. All but five of the individuals in a risk group receiving two doses were documented to be immunocompromised. The most common risk factor was a chronic respiratory condition (228 individuals), while liver condition was the rarest with only 9 individuals. The highest proportion of individuals with pre-vaccine 2009 pandemic H1N1 HI antibody titres ≥1∶32 were found in healthy children <5 years (27%) and young adults (10 to 39 years) in a risk group (33%).

**Table 1:** Summary of cohort for analysis.

Variable	Level	<5 years (n = 60)	Pregnant only(n = 32)	Risk group 1 dose(n = 456)	Risk group 2 dose(n = 62)
Priority group	Pregnant	0	32	4	0
	Chronic disease	4	0	441	5
	Immunocompromised	0	0	4	8
	Chronic disease & Immunocompromised	0	0	11	49
Age	<5	60	0	0	0
	10 to 39	0	32	54	9
	40 to 54	0	0	107	23
	55 to 64	0	0	168	21
	65+	0	0	127	9
Sex	Male	31	0	220	28
	Female	29	32	236	34
Ethnicity	White	51	29	446	60
	Non-White	9	3	10	2
2007/08 Seasonal vaccine	No	60	32	119	27
	Yes	0	0	337	35
					
2008/09 Seasonal vaccine	No	60	32	89	18
	Yes	0	0	367	44
					
2009/10 Seasonal vaccine	No	60	29	39	7
	Yes	0	3	417	55
Vaccination month	Nov	0	21	397	60
	Dec	0	3	59	2
	Jan	11	2	0	0
	Feb	40	6	0	0
	March	9	0	0	0
ILI (Between vaccinationand post vaccination blood sample)	No	57	31	414	56
	Yes (≤7 days from vaccine)	2	1	31	4
	Yes (>7 days from vaccine)	1	0	11	2
Interval to post vaccinationblood sample	14–20 days	7	3	44	2
	21–27 days	35	17	148	30
	28–34 days	15	6	62	11
	35–41 days	2	0	16	14
	42–48 days	0	1	14	4
	49–55 days	1	4	51	1
	56–62 days	0	0	65	0
	63–69 days	0	0	42	0
	70–87 days	0	1	14	0

The seroconversion rate in healthy children aged <5 years (87%) was higher than that of all risk groups combined (65%) (p<0.001) (Table 2). There was also a trend towards a higher seroconversion rate in pregnant women (81%) than in risk groups although this did not reach significance (p = 0.08). Those who received two doses showed a higher seroconversion rate compared to those given one dose but the difference was not significant (Immunocompromised with one dose = 53% v two doses = 68%, p = 0.36). There was no difference in immune response by individual risk group so all groups were combined for subsequent analysis.

**Table 2:** Summary of vaccine responses.

Group	N	Pre-vaccine≥1∶32n (%)	Post-vaccineseroprotection n (%)	Post-vaccineseroconversionn (%), [95% CI]	Post vaccination GMT(95% CI)	GMFR (95% CI)
<5 years	60	16 (27%)	55 (92%)	52 (87%)[75%–94%]	185(117–294)	15.9(11.9–21.3)
Pregnant	32	6 (19%)	28 (88%)	26 (81%)[64%–93%]	154(80–297)	21.2(10.8–41.6)
Risk (1 dose)	456	76 (17%)	335 (73%)	293 (64%)[60%–69%]	68(59–79)	9.0(7.8–10.3)
Risk (2 dose)	62	14 (23%)	53 (85%)	44 (71%)[58%–82%]	120(79–183)	12.5(8.1–19.4)
Risk (all)	518	90 (17%)	388 (75%)	337 (65%)[61%–69%]	73(63–84)	9.3(8.1–10.7)

**Figure 1:**
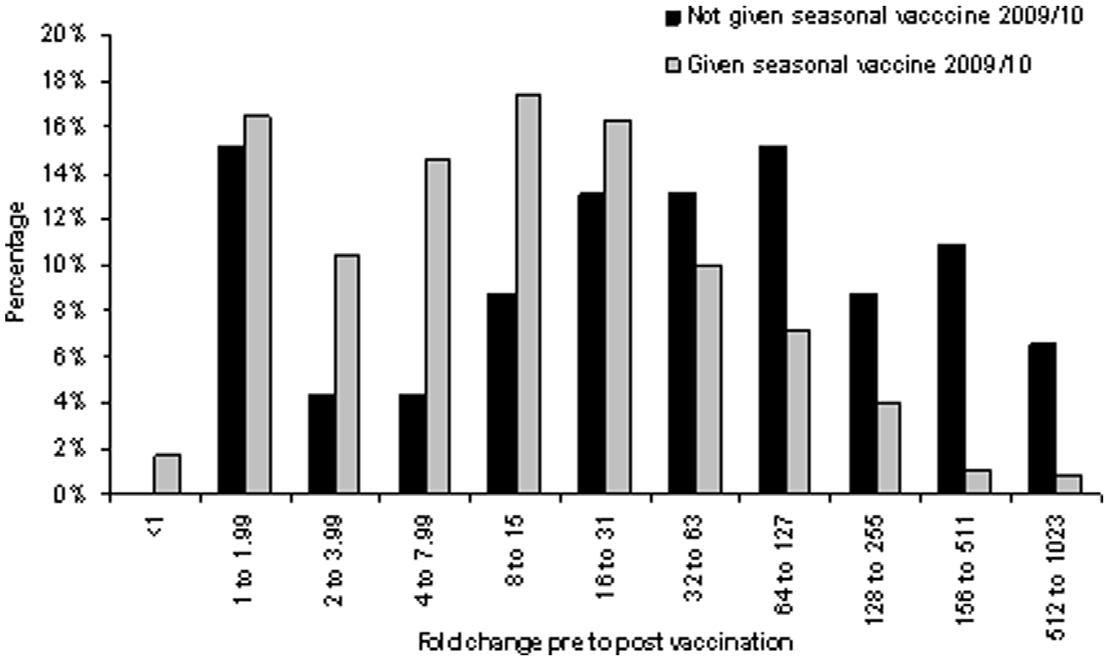
Distribution of fold responses pre to post Pandemrix™ vaccination by 2009 seasonal influenza vaccination status.

Multivariable analysis of the seroconversion rate in healthy children <5 years showed that there was a significant age effect when stratifying into <2.5y (94%) and 2.5y to 5y (79%) (p = 0.03). In the pregnant group, no significant factors were associated with seroconversion. In risk groups, there was no effect of individual disease type, sex, ethnicity, or month of blood collection. There was evidence of lower seroconversion rates in the oldest age group (Table 3). The size of the response in those who did respond was found to decrease as the pre-vaccination titre increased, indicating a possible threshold effect. Individuals who reported an ILI shortly after vaccination were found to have higher seroconversion rates (OR: 4.19, 95% CI:1.40 to 12.53). Interval between vaccination and blood collection had a significant effect on size of response in those who seroconverted.with a 5% drop per week as antibodies waned. Receipt of seasonal TIV in one year was correlated to other years which made assessment of the independent effects of each year less powerful. The model was therefore fitted with all three years included (as shown in table 3) and also with each year included without the other years. This showed that whilst history of seasonal TIV for the seasons 2007/08, 2008/09 or 2009/10 did not have a significant effect on seroconversion rate, the size of response in those who did seroconvert was lower in those who received the 2009/10 seasonal TIV (Fold effect 0.52, 0.35 to 0.78), as illustrated in figure 1 and shown in Table 3.

**Table 3:** Multivariable model of seroconversion within those vaccinated in risk groups (chronic disease and/or immunocompromised).

			Seroconversion		Fold ratio (post:pre) if seroconverted	
Variable	Level	n/N withseroconversion (%)	OR (95% CI)	p-value	Fold effect	p-value
Pre-vaccine HIantibody level	<8	245/364 (67%)	1.00		1.00	
	8–31	41/64 (64%)	0.95 (0.54–1.67)	0.86	0.66 (0.47–0.91)	0.01
	32–127	44/67 (66%)	0.91 (0.51–1.61)	0.74	0.33 (0.24–0.45)	<0.001
	128+	7/23 (30%)	0.18 (0.07–0.49)	0.001	0.15 (0.07–0.32)	<0.001
Age	10–39	46/63 (73%)	1.00		1.00	
	40–54	94/130 (72%)	0.78 (0.37–1.66)	0.52	0.82 (0.57–1.18)	0.28
	55–64	126/189 (67%)	0.64 (0.31–1.32)	0.23	0.92 (0.64–1.31)	0.64
	65+	71/136 (52%)	0.42 (0.20–0.89)	0.03	0.68 (0.46–1.02)	0.06
ILI (Betweenvaccination and postvaccination blood sample)	No	299/470 (64%)	1.00		1.00	
	≤7 days from vaccine	31/35 (89%)	4.19(1.40–12.53)	0.01	0.96(0.67–1.38)	0.83
	>7 days from vaccine	7/13 (54%)	0.72(0.22–2.33)	0.57	1.86(0.88–3.92)	0.10
Doses	1	293/456 (64%)	1.00		1.00	
	2	44/62 (71%)	1.26 (0.68–2.37)	0.46	1.10 (0.80–1.52)	0.54
Interval to post vaccination Blood sample	Weeks	continuous	0.99 (0.91–1.07)	0.72	0.95 (0.91–0.99)	0.03
2007/08 seasonalvaccine	No	111/146 (76%)	1.00		1.00	
	Yes	226/372 (61%)	0.69 (0.39–1.23)	0.21	0.80 (0.60–1.07)	0.14
2008/09 seasonalvaccine	No	81/107 (76%)	1.00		1.00	
	Yes	256/411 (62%)	0.99(0.51–1.88)	0.94	0.90(0.65–1.25)	0.54
2009/10 seasonalvaccine	No	36/46 (78%)	1.00		1.00	
	Yes	301/472 (64%)	0.78(0.33–1.85)	0.58	0.52(0.35–0.78)	0.002

Logistic regression modelling with 2009 pandemic H1N1 pre-vaccine titres ≥1∶32 as an outcome in healthy children <5 years and pregnant women group did not identify any predictive factors. In risk groups, only age was significantly associated (p = 0.003) with the 10 to 39 years age group more likely to have pre-vaccine tires ≥1∶32. There was also some evidence that prior seasonal TIV (any year from 2007–2010) may have an association as 18.2% (85/466) of those with a history of seasonal TIV had pre-vaccination titres ≥1∶32 compared to 9.6% (5/52) with no history (p = 0.04 adjusting for age).

## Discussion

The immunogenicity of oil in water ASO3 adjuvanted 2009 pandemic influenza H1N1 vaccine (Pandemrix™) in those with chronic disease or immunocompromised, pregnant women and healthy children <5 years met European Medicines Agency licensing criteria but in some groups was lower than expected compared to limited published data in risk groups. [11], [12], [13] The highest antibody response was seen in children <5 years and pregnant women; among those with chronic conditions immunogenicity declined with age (Table 3). These immunogenicity findings are consistent with the clinical protection observed in these groups from the same population reported by Andrews et al who found that efficacy was highest in healthy children under 5 years of age and young adults (77% to 100%), declining with age to 22% (−153% to 76%) in those aged 25–49-year with chronic conditions. [6].

Receipt of seasonal TIV in previous seasons (2007/8 to 2009/10) had no significant effect on seroconversion rate. However, receipt of the 2009/2010 seasonal TIV resulted in a negative effect on the size of the response in persons in risk groups who showed around half the fold change compared to non-recipients of this particular seasonal influenza vaccine. A negative effect on AS03 adjuvanted 2009 pandemic influenza H1N1 vaccine (PandemrixTM) immune response size associated with prior receipt of seasonal TIV had previously only been reported in studies involving healthy children, adult volunteers and elderly. [4], [5], [13] Other studies have reported such effects in healthy volunteers correlated to history of seasonal TIV in previous years. [14], [15], [16] The explanation for these observations remains obscure. In many other vaccines, the amount of antibodies produced is related to the level of clinical protection. Modelling studies on Influenza HI antibodies suggest that higher levels of HI antibodies may be correlated with better clinical protection and the current use of a cut off threshold may not be appropriate. [17], [18] Although it is unclear what effect such reductions have on protection *in vivo*, our findings would suggest a possible link. The quantity of antibody generated may be only a concern for young adults in risk groups and not the elderly who despite lower seroconversion rates, tend to produce antibodies of significantly superior quality (repertoire and avidity) due to the presence of long term memory B-cells following exposure before 1957. [19] Furthermore, T cell response were found to be greater post pandemic H1N1 2009 vaccination in the elderly ≥60 years and challenge studies have shown that pre-existing CD4+ T cells may be associated with influenza protection. [20], [21].

We did not find any significant difference in seroconversion rate by risk group, underlying disease condition or number of 2009 pandemic H1N1 vaccine doses received. Interestingly, individuals who report influenza like illness symptoms within 7 days of receiving the vaccine were more likely to seroconvert. It is unlikely that ILI reports are the result of infection since there was no correlation between interval to post-vaccination blood sample with seroconversion rate. A study of children aged between 9 months and 10 years who received either the same oil-in water AS03-adjuvanted split virion or a non-adjuvanted whole virion H1N1 (2009) vaccine found that children who reported a fever ≥38°C after vaccination demonstrated higher HI titres than those that did not. [4] These signs and symptoms could be the result of cytokine release from peripheral blood mononuclear cells very quickly after vaccination and are predictors of good immune responders in both children and adults with chronic disease.

The pre-vaccination seropositive rates were similar in both children <5 years (27%) and risk groups aged 10 to 39 years (33%) in our cohort. However, predicted immunological boosting after 2009 pandemic H1N1 vaccination only occurred in children (seroconversion rate = 92%) and not in adolescents and young adults from risk groups after excluding those receiving 2 doses because of immunocompromised status (Age 10 to 39 seroconversion rate = 74%) Furthermore, in risk groups, pre-vaccine titre level was an important negative predictor of the size of the HI antibody response (fold changes) at all levels above 1∶8 (Table 3). The reason for these different immune responses is difficult to decipher. Seasonal TIV is not part of the routine schedule for healthy children <5 years in UK and hence pre-vaccine titres are the result of natural pandemic H1N1 infection only. [22] However, the source of pre-vaccine titres in adolescents and young adults in risk groups will include the additional effects of previous seasonal influenza vaccination and the generation of cross reactive antibodies. Whether, the reason for this observation is specific to a difference in the origins of the pre-existing antibodies or specific to the groups; the finding clearly highlights the limitations of extrapolating results from influenza vaccine studies of healthy individuals to risk groups with history of seasonal TIV. The possible role of these pre-vaccination antibodies on clinical protection or lack of it in individuals with chronic conditions requires further research.

Our opportunistic recruitment method is unlikely to substantially affect the outcomes investigated but we cannot rule out the effect of unknown confounders resulting from selection bias. Laboratory colleagues carrying out the testing did not have access to any epidemiological data of participants such as age or seasonal vaccination status. It may be possible that for some individuals details of seasonal vaccination histories may have been incorrect (i.e. were lost or not available for individuals who had just recently joined a general practice especially if they had moved from abroad). However, such misclassification could have occurred for any previous seasonal influenza vaccination history and should therefore not affect the findings related to differences between the different vaccine years. The proportion classified as having received seasonal influenza vaccination in each of the three seasons, 61% in 2007, 67% in 2008 and 77% 2009 is very similar to routine seasonal influenza coverage of about 70% in those aged 65+ years and about 45% in those <65 years belonging to risk groups, lending further support to the representativeness of our sample set. [23] Finally, we have looked at HI titres in this study which mainly represent antibodies that inhibit viral haemagglutinin binding to host cells. Studies of HI titres as surrogate marker for vaccine immunogenicity and correlates of protection were based on healthy individuals. [24], [25], [26] However, our results did show a correlation between reduced HI antibody response with reduced clinical protection and vice versa. Finally, we were unable to study cell mediated immunity or investigate differences in avidity of the antibodies generated.

### Conclusion

We have found that seroresponse to an oil-in water adjuvanted 2009 pandemic H1N1 vaccine was lower than expected in subjects from risk group with chronic disease.

Receipt of 2009/10 seasonal TIV resulted in a reduction in the size of the pandemic 2009 H1N1 adjuvanted vaccine response by as much as 50% in individuals with chronic disease. Our findings of reduced immunogenicity in ‘high risk’ individuals correlates with the lack of clinical protection identified in young adults in these groups from vaccine effectiveness study of the same population in England. Further research to understand this is critical for future pandemic vaccine development and policies.

## Acknowledgments

We would like to express our sincere appreciation to Pauline Kaye, Deborah Cohen, Teresa Gibbs and Vaccine research nurses of Hertfordshire and Gloucestershire for their support and dedication to the project. We would also like to thank the participants for volunteering to take part in the evaluation. We are grateful for the technical support provided by staff at the Respiratory Virus Unit: Janice Baldevarona, Surita Gangar, Justine Candy and Paul Kotzampaltiris.
